# Polypyrrole-Modified Nylon 6 Nanofibers as Adsorbent for the Extraction of Two β-Lactam Antibiotics in Water Followed by Determination with Capillary Electrophoresis

**DOI:** 10.3390/molecules24122198

**Published:** 2019-06-12

**Authors:** Xinghua Li, Junjie Miao, Zhendong Yin, Xiangdong Xu, Hongmei Shi

**Affiliations:** School of Public Health, and Key Laboratory of Environment and Human Health of Hebei Province, Hebei Medical University, Shijiazhuang 050017, China; lixh026@163.com (X.L.); miao4820@163.com (J.M.); zhendongyin123@163.com (Z.Y.); xuxd@hebmu.edu.cn (X.X.)

**Keywords:** PPy-PA6-NFsM, β-lactam residues, solid phase extraction, determination, CE-DAD

## Abstract

A solid phase membrane adsorbent—a nylon 6 nanofibers membrane coated by polypyrrole (PPy-PA6-NFsM)—was firstly synthesized and used for extraction of two β-lactam antibiotics (oxacillin and cloxacillin) in urban river water. Then the analytes were detected by capillary electrophoresis with a diode array detector (CE-DAD). The synthesized nanofibers membrane was characterized by scanning electron microscopy and a Fourier transform infrared spectrometer. The experimental conditions were optimized, including the amount used of PPy-PA6-NFsM, pH of the sample solutions, adsorption volume, and desorption conditions. Under the optimal extraction and separation conditions, the detection limits were found to be 2.0 ng/mL for both oxacillin and cloxacillin. The proposed method was applied to the determination of the two β-lactams in water samples of an urban river. The recoveries of these two β-lactams were found to be in the range 84.2–96.4%, demonstrating that PPy-PA6-NFsM has a high extraction capability for these two antibiotics. The relative standard deviations, ranging from 2.26% to 5.29% for intraday measurements and from 2.38% to 7.02% for inter-day determinations, were derived respectively.

## 1. Introduction

Antibiotics are an important group of pharmaceuticals and personal care products, which have been extensively used in human therapies and veterinary medications, as well as in aquaculture [1]. Incomplete removal and improper waste disposal can lead to the release of these excreted antibiotics into the aquatic environment by various pathways [2]. On account of continuous introduction into the environment and permanent presence, antibiotics are regarded as “pseudo persistent compounds” [3]. Increasing attention has been paid to the generation and environmental spread of antibiotic resistance genes (AGRs) due to overuse of various antibiotics [4]. β-lactam antibiotics have been used in treatments of a variety of bacterial infections, including gram-positive bacteria as well as some gram-negative bacteria [5]. In most countries, β-lactam antibiotics make up approximately 50–70% of total antibiotic use in human beings [6]. Recently, several studies have shown that there were different levels of β-lactam antibiotic residues in different water environments. These studies showed that amoxicillin and penicillin were detected in wastewater influents at concentrations ranging from 114.5 to 586.3 ng/L and 13.2 to 65.89 ng/L, while the concentrations were found for amoxicillin and penicillin in wastewater effluents ranging from 24.54 to 97.40 ng/L and <LOQ (limit of quantification) to 31.18 ng/L. Amoxicillin was detected in the river at the lowest concentration of 15.55 ng/L, but the lowest concentration of penicillin was at a concentration of <LOQ [7,8].

Capillary electrophoresis (CE) has been developed rapidly in recent years because of its advantages including high speed, a separation efficiency, and automation [9]. However, the low detection sensitivity is its intrinsic disadvantage when the UV detector is used. More sensitive detectors including mass spectrometry (MS) [10,11] and laser-induced fluorescence (LIF) [12,13] have been applied to improve the detection sensitivity, but these detectors are relatively expensive for purchase and maintenance.

Enrichment of target material by a pretreatment is often a reliable method to improve detecting sensitivities. Solid phase extraction (SPE) is the most commonly used sample pretreatment technique for purifying and concentrating trace contaminants [7,14,15,16]. In recent years, new types of materials have been in focus for making highly efficient SPE adsorbents [17]. Electrospun nanofibers are widely applied in many fields, such as biosensors and supercapacitor electrodes [18,19]. Nanofibers as adsorbents have been a research interest due to their remarkably adsorptive capacity, controllable size, and functionality [14]. Such kind of adsorbents were used for removal of toxic dyes and arsenite from aqueous solutions [20,21], and extraction of steroids and non-steroidal anti-inflammatory drugs from water samples [22,23]. They were also applied for extraction of cortisol from human hair [24]. Nylon 6 nanofibers membrane (PA6-NFsM) is particularly effective for extraction of non-polar and medium-polar compounds in aqueous samples [25,26]. The properties of PA6-NFsM can be modified to confer adsorption of different analytes.

There has been a growing interest in intrinsic conducting polymers with conjugated double bonds as adsorbents or as coating nanomaterials [27,28]. For example, polypyrrole (PPy) can interact with target molecules in various forms because of its non-localized π electron conjugate systems and multifunctional properties. These properties lead to intermolecular interactions like acid-base, hydrogen bonding and exchange between the analytes and polymer [29]. Also, it has an excellent chemical stability in neutral and acidic media [30]. PPy can be coated on electrospun nanofibers by chemical deposition [31]. Recently, PPy as a coating nanomaterial for SPE has been utilized to remove pollutants from aquatic media [31,32,33,34,35,36,37]. Also, it has been applied successfully to solid-phase microextraction of chemotherapeutics, antidepressants, and antihypertensive drugs in biological samples [25,38,39,40]. However, its applications to the extraction of trace antibiotics in environmental waters remain to be explored.

**Figure 1:**
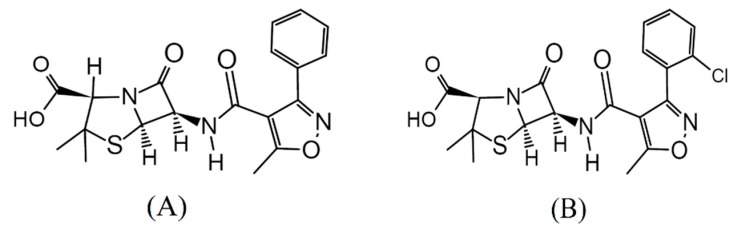
Structure of oxacillin (**A**) and cloxacillin (**B**).

In this work, PPy has been coated on PA6-NFsM and the modified nanomaterial (PPy-PA6-NFsM) for extraction of oxacillin (OXA) and cloxacillin (CLOX) from urban water samples has been exploited (structures of OXA and CLOX are illustrated in Figure 1). Factors affecting the extraction efficiency, including the amount of PPy-PA6-NFsM, the pH values of sample solutions, extraction time, and desorption conditions, have been investigated. The established method has been applied for the determination of the two antibiotics in actual water samples. We herein report these results.

## 2. Results

### 2.1. Characterization of PPy-PA6-NFsM

**Figure 2:**
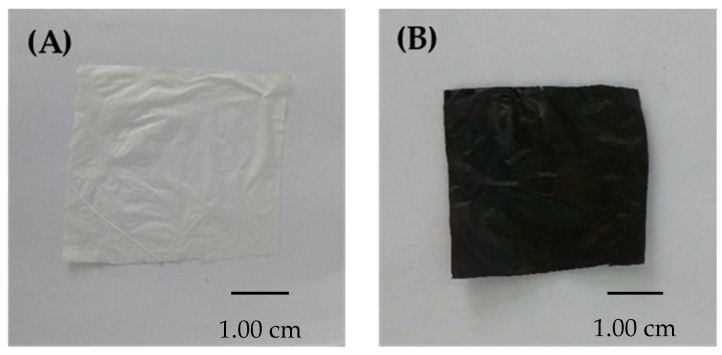
The picture of nylon 6 nanofibers membrane (PA6-NFsM) (**A**) and PPy/PA6-NFsM (**B**).

**Figure 3:**
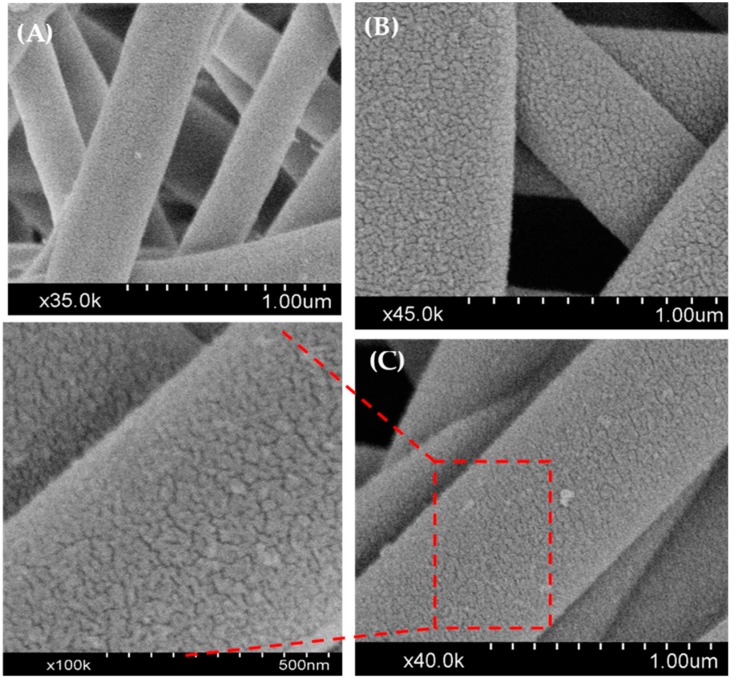
SEM images of PA6-NFsM (**A**) and PPy-PA6-NFsM before (**B**) and after (**C**) extraction of β-lactam antibiotics.

As is shown in Figure 2, PA6-NFsM is white. After modification with PPy, PPy-PA6-NFsM is black. Figure 3 shows the SEM images of PA6-NFsM, as shown in Figure 3A, and PPy-PA6-NFsM, as shown in Figure 3B. Compared to PA6-NFsM, the surface of PPy-PA6-NFsM was relatively rough. In addition, the diameters of PPy-PA6-NFsM were increased significantly compared to that of PA6-NFsM. After measurement and estimation, the diameter of PA6-NFsM was about 447 ± 64 nm, while the diameter of PPy-PA6-NFsM was about 709 ± 90 nm. The images reveal that PPy completely covers the surfaces of PA6-NFsM. Figure 3C is the SEM image of PPy-PA6-NFsM after extraction of the analytes. It was found that some particles were attached to the surface of PPy-PA6-NFsM. These particles were considered as the analytes after drying.

**Figure 4:**
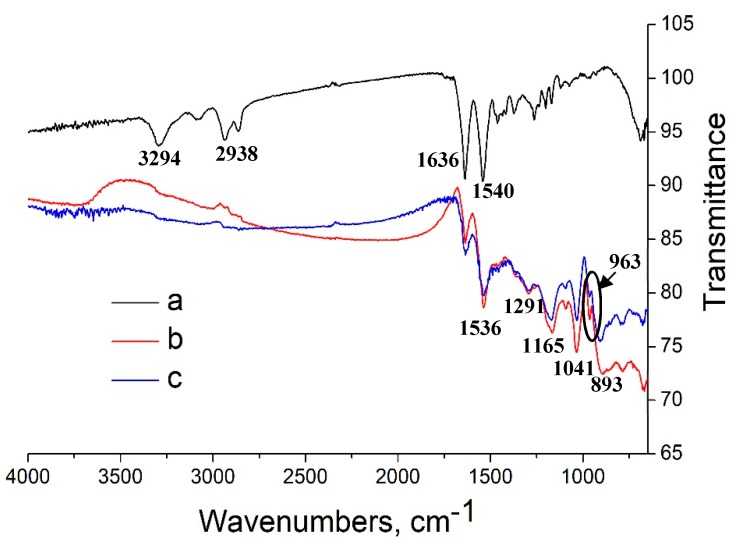
FTIR spectra of PA6-NFsM (**a**) and PPy-PA6-NFsM before (**b**) and after (**c**) extraction of β-lactam antibiotics.

Figure 4 shows the FTIR spectra for the two types of nanofibers. Characteristic absorption bands of PA6-NFsM, as shown in Figure 4a, are as follows—the coupled motions of C=O stretching and N-H in plane bending at 1500–1700 cm^−1^ and the N–H and C–H stretching vibrations at 2800–3300 cm^−1^ [41]. Compared to PA6-NFsM, absorption bands of PPy-PA6-NFsM, as shown in Figure 4b, were changed; C=O, N-H, and C-H stretching vibrations all decreased. Absorption bands from PPy, including C–N stretching at 1165 cm^−1^, C–H stretching at 963 cm^−1^, and C–H deformation at 893 cm^−1^, all appeared in the final material. The peak at 1536 cm^−1^ was from the C=C stretching of the pyrrole ring, and the peaks at 1291 and 1041 cm^−1^ were related to the in-plane vibration and in-plane bending of C–H [42]. The broad band in the region between 1600 up to 3000 cm^−1^ was characteristic of the conducting form of PPy [43]. These results support that PPy was coated successfully on the surfaces of PA6-NFsM. The spectra of PPy-PA6-NFsM after the adsorption process is displayed in Figure 4c. Compared with the spectra before the adsorption process, the intensity of the characteristic absorption peaks of PPy was weakened.

### 2.2. Optimization of the Conditions for Capillary Electrophoresis with a Diode Array Detector (CE-DAD) Analysis

**Figure 5:**
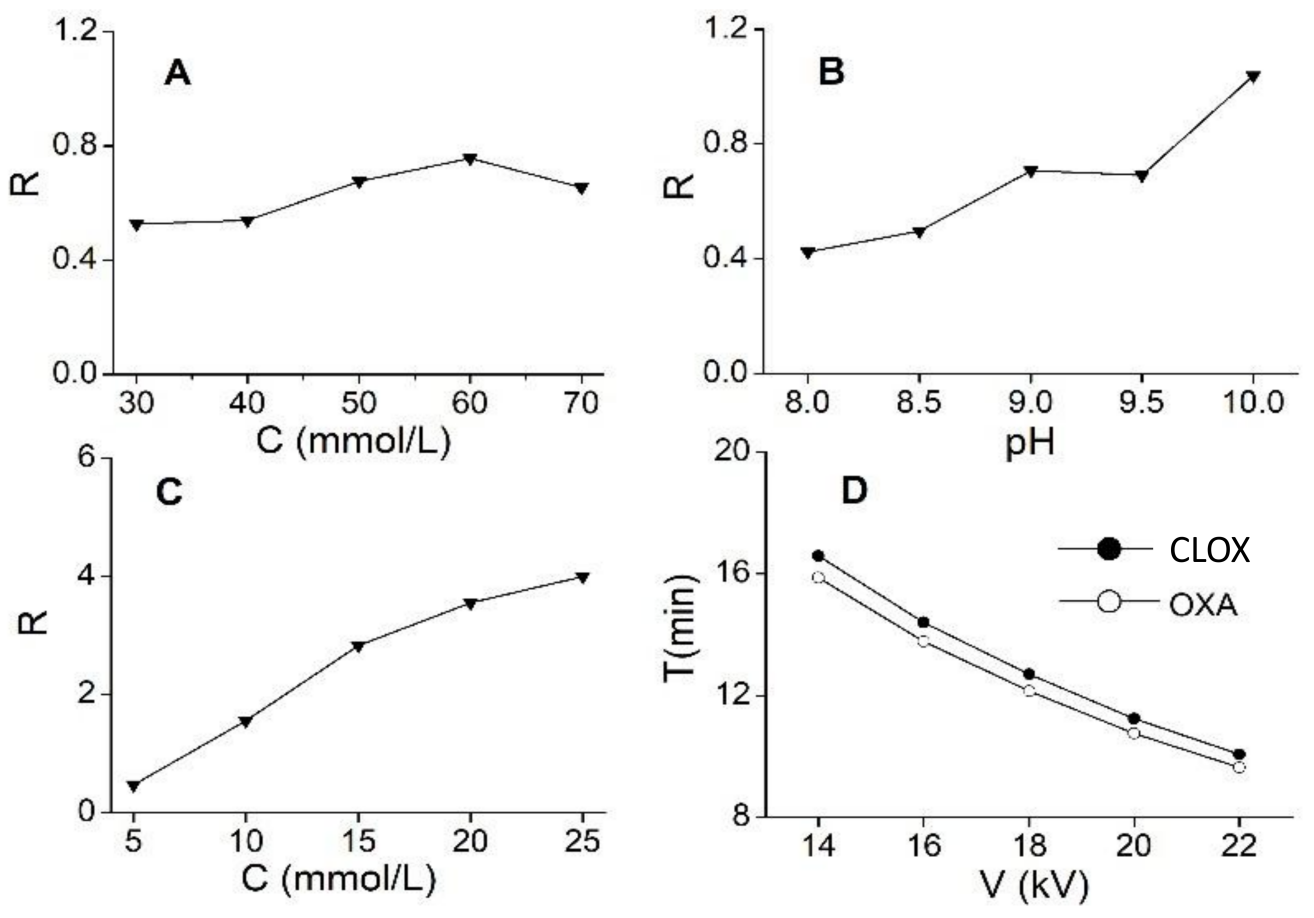
Effect of parameters of capillary electrophoresis with a diode array detector (CE-DAD) on the separation of analytes. Conditions: (**A**) the concentration of borax in the buffer; (**B**) the pH of the buffer; (**C**) the concentration of sodium dodecyl sulfate (SDS) in the buffer; (**D**) the separation voltage. R, the resolution between oxacillin (OXA) and cloxacillin (CLOX); T, the migration time of analytes. The concentration of analytes: 5.0 μg/mL.

The parameters affecting the separation of the two β-lactams were investigated, such as the pH, the concentration of borax and sodium dodecyl sulfate (SDS) in the buffer solution, and the separation voltage. In CE, the resolution (*Rs*) is also used as an indicator of the separation efficiency of the capillary. The expression for calculating *Rs* is as follows:

(1) $$R_{s}=\frac{2\times \left(t_{2}-t_{1}\right)}{\omega_{2}+\omega_{1}}$$

where *t*_1_ and *t*_2_ represent the migration time of any two adjacent peaks (*t*_2_ > *t*_1_), ω_1_ and ω_2_ are the peak widths of two adjacent peaks. The migration time of each analyte was positively correlated with the concentration of borax and SDS in the buffer solution and the pH of the buffer, while the migration time was negatively correlated with the separation voltage. As is shown in Figure 5, when pH was 10.0, a higher resolution was obtained, but the migration time of CLOX was similar to the solvent peak. Therefore, considering the resolution and the migration time, the optimal separation conditions are as follows—when the running buffer consisted of 60.0 mmol/L borax and 15.0 mmol/L SDS (pH 9.0) and the separation voltage was 20 kV, the resolution of the two β-lactams could meet the quantitative needs (*Rs* > 1.5).

### 2.3. Optimization of the Conditions for Solid Phase Membrane Extraction

#### 2.3.1. Effect of the Amount of PPy-PA6-NFsM

**Figure 6:**
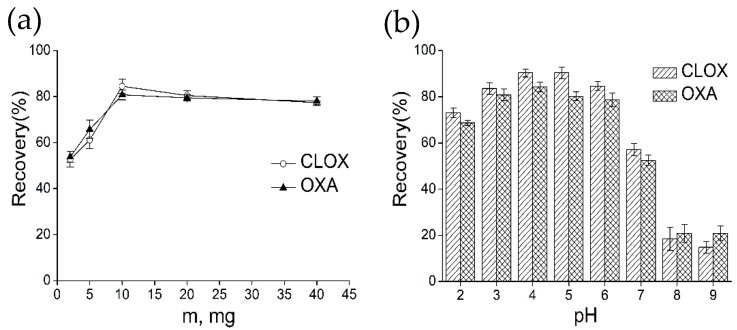
Effect of the amount of PPy-PA6-NFsM (**a**) and pH (**b**) on the recoveries of analytes. Conditions: sample volume, 20.0 mL; concentration of two analytes, 0.5 μg/mL; extraction time, 30 min; eluent, 2.0 mL of 1% ammonia/ACN (*v*/*v*); desorption time, 30 min.

The recoveries of the two β-lactams were investigated when the amount of PPy-PA6-NFsM were varied from 2.0 to 40.0 mg, as shown in Figure 6a. Recoveries increased gradually with an increase in the amount from 2.0 to 10.0 mg and decreased slightly from 10.0 to 40.0 mg. When the volume of the eluent was fixed, and a large amount of adsorbent was used, the target antibiotics could not be completely eluted from PPy-PA6-NFsM. Thus, 10.0 mg of PPy-PA6-NFsM was chosen for the subsequent experiments.

#### 2.3.2. Effect of Solution pH

The pH is a key factor affecting extraction of β-lactams by PPy-PA6-NFsM since the molecular states of the β-lactams were affected by the solution pH. The effect of pH change on the extraction was investigated in a range of 2.0 to 9.0, as shown in Figure 6b. The extraction efficiency under acidic conditions was better than under the neutral and basic ones. The recoveries were achieved with the highest in the pH range of 3.0 to 6.0. In this pH range, the two β-lactams are likely in the form of anions since the pKa values of β-lactams are between 2.5 and 2.8. In this pH range, PPy took the cationic form, so that the β-lactams were absorbed onto PPy-PA6-NFsM via charge attraction. A solution pH 4.0 was thus selected.

#### 2.3.3. Effect of Extraction Time

**Figure 7:**
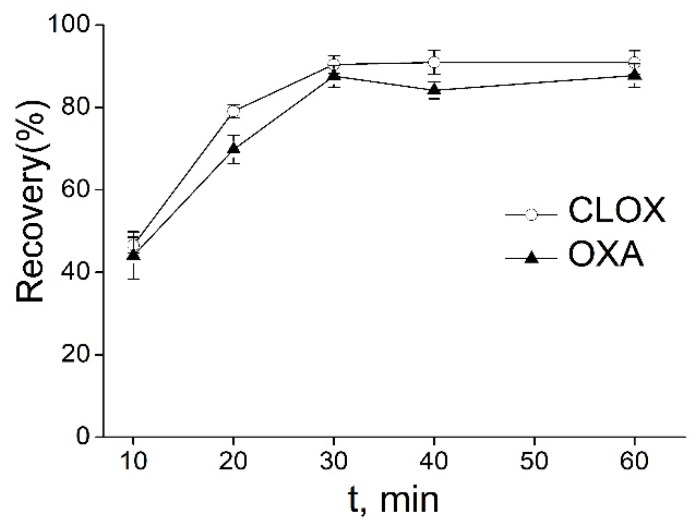
Effect of the extraction time on the recoveries of analytes. Conditions: the amount of PPy-PA6-NFsM, 10.0 mg; sample volume, 20.0 mL; concentration of two analytes, 0.5 μg/mL; pH, 4.0; eluent, 2.0 mL of 1% ammonia/ACN (*v*/*v*); desorption time, 30 min.

The stirring time was increased from 10 to 60 min, allowing for examination of the time effect. The results displayed in Figure 7 indicate that the adsorption equilibrium was achieved in 30 min and slightly changed when the extraction time became longer. Consequently, the extraction time was chosen as 30 min.

#### 2.3.4. Optimization of Desorption Conditions

**Figure 8:**
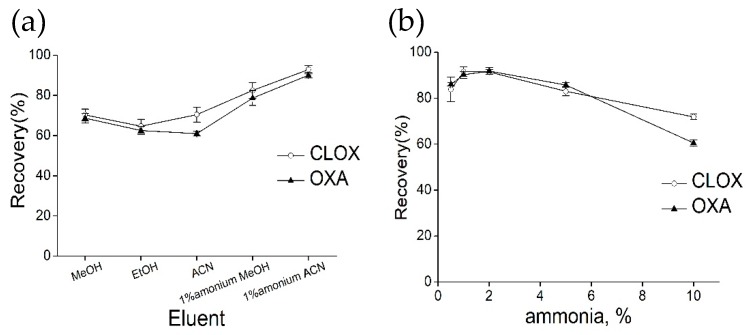

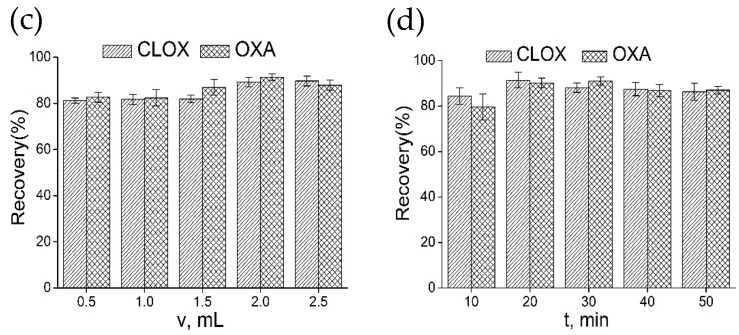
Optimization of the desorption conditions on the recoveries of analytes. (**a**) Effect of elution solvent; (**b**) effect of the content of ammonia in eluent; (**c**) effect of eluent volume; (**d**) effect of desorption time. Conditions: the amount of PPy-PA6-NFsM, 10.0 mg; sample volume, 20.0 mL; concentration of two analytes, 0.5 μg/mL; pH, 4.0.

Methanol (MeOH), ethanol (EtOH), acetonitrile (ACN), 1% ammonia/MeOH (*v*/*v*), and 1% ammonia/ACN (*v*/*v*) were investigated as eluent. The results are shown in Figure 8a–d. Clearly, 1% ammonia/ACN was the best eluent in terms of desorption efficiency. Moreover, the content of ammonia in ACN (0.5%, 1%, 2%, 5%, 10%) as eluent was also studied. Satisfactory recoveries were obtained in eluent containing less than 2% ammonia. In addition, the elution volume and desorption time were also studied. The condition of using 2.0 mL of 2% ammonia/ACN to shake for 30 min under a shaker rate of 400 rpm was selected.

#### 2.3.5. Extraction Volume

**Figure 9:**
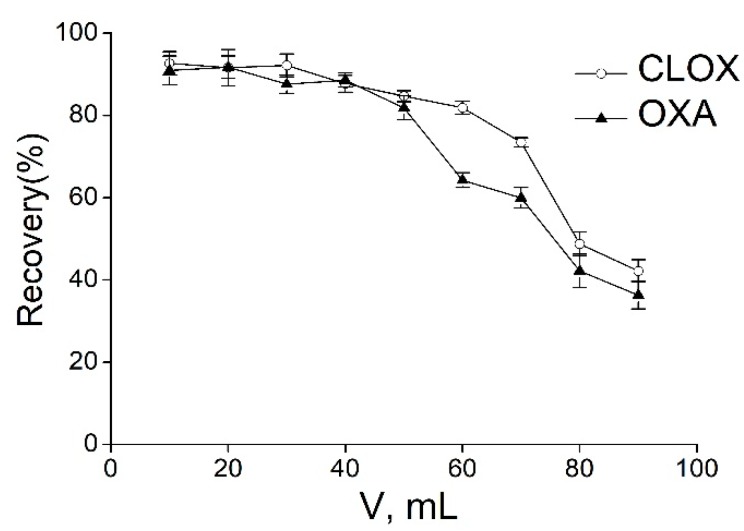
Effect of the extraction volume on the recoveries of analytes. Conditions: the amount of PPy-PA6-NFsM, 10.0 mg; concentration of two analytes, 0.5 μg/mL; pH, 4.0; eluent, 2.0 mL of 2% ammonia/ACN (*v*/*v*); desorption time, 30 min.

The sample volume was varied from 10.0 to 90.0 mL in order to optimize the recovery. The results are illustrated in Figure 9. When the sample volume gradually increased in the range 10.0–40.0 mL, the recovery rate remained above 80%. As the sample volume continued to increase, the recovery decreased obviously. A sample volume of 40.0 mL was chosen as the extraction volume.

### 2.4. Reusability of PPy-PA6-NFsM

**Figure 10:**
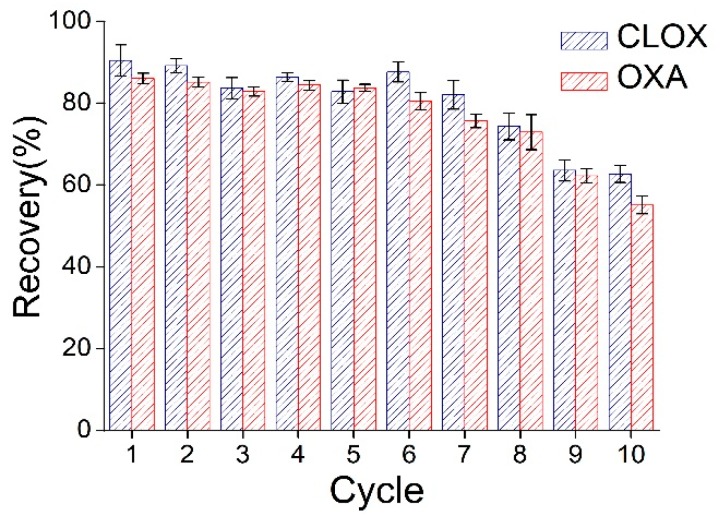
Reusability study: recovery of two β-lactams depending on the number of cycles of adsorption–desorption. Conditions: the amount of PPy-PA6-NFsM, 10.0 mg; sample volume, 40.0 mL; concentration of two analytes, 0.5 μg/mL; pH, 4.0; eluent, 2.0 mL of 2% ammonia in acetonitrile (*v*/*v*); desorption time, 30 min.

The reusability of PPy-PA6-NFsM was also investigated; it was washed twice, each time with 5.0 mL of 2% ammonia/ACN and 5.0 mL water after the desorption of the analytes, and then it was reused for the next extraction cycle of the β-lactams. The results in Figure 10 demonstrate that PPy-PA6-NFsM can be reused at least five times while retaining its efficiency.

### 2.5. Analytical Performance

The parameters obtained for the analytical performance of the proposed method are depicted in Table 1. Under the optimized conditions, the linearity range, limit of detection (LOD) and limit of quantification (LOQ) were calculated. The values of LOD, and LOQ were calculated as the concentration corresponding to 3 and 10 times above the signal-to-noise ratios. A good linearity was obtained in the range 5.0–250.0 ng/mL for both OXA and CLOX. The LOD and LOQ for the two β-lactams were 2.0 and 5.0 ng/mL, respectively. The LOD and LOQ obtained without PPy-PA6-NFsM extraction for the two β-lactams were 0.1 and 0.2 μg/mL, respectively. In contrast, the method with PPy-PA6-NFsM extraction could obtain a lower LOD.

### 2.6. Comparison with Other Methods

Comparison of various methods for the determination of the two β-lactams is in Table 2. Experiments with traditional solid phase extraction require repeated loading procedures and are cumbersome to operate. Compared with other methods, the described method is relatively simple. In addition, the loss in SPE procedure and other steps was limited, and time for pretreatment was saved.

### 2.7. Application to Real Samples

**Figure 11:**
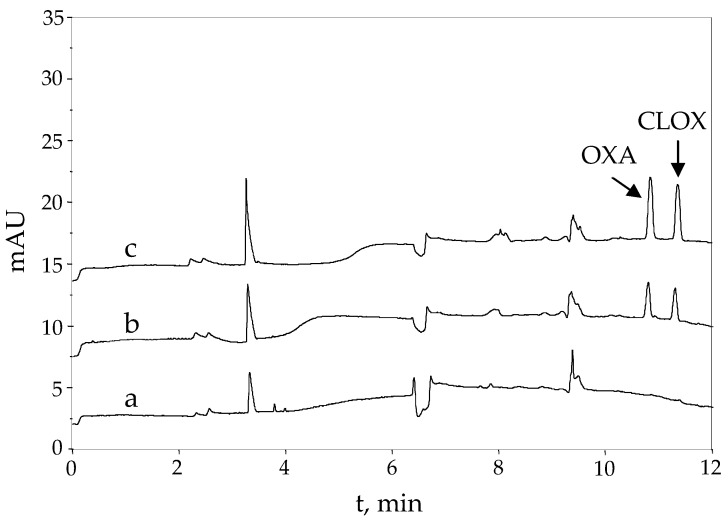
Electropherograms of the blank water sample (**a**), the blank river water sample spiked with 50 ng/mL (**b**), the blank river water sample spiked with 100.0 ng/mL (**c**). Conditions: the amount of PPy-PA6-NFsM, 10.0 mg; sample volume, 40.0 mL; sample pH, 4.0; extraction time, 30 min; eluent, 2.0 mL of 2% ammonia/ACN (*v*/*v*); desorption time, 30 min.

The established method was applied to the determination of the two β-lactams in real water samples acquired from three different areas of Minxin River. Several pharmaceutical companies are located along Minxin River. Three sampling points are set on the up (sample 1), middle (sample 2), and low (sample 3) parts of the river. Under the optimized conditions, the real samples were extracted with the PPy-PA6-NFsM, followed by CE-DAD analyses. The results showed that these two antibiotics could not be detected in any one of the samples. A possible explanation is that the concentrations of the antibiotic residues in water samples were below the detection limits. To evaluate accuracy of the proposed method at different concentration levels of the analytes, three different concentrations of the analytes were spiked in the river water samples. Under optimal conditions, the electropherograms are displayed in Figure 11 showing good separations. As listed in Table 3, recoveries were found to be in the range 84.2–96.4%. Moreover, the intraday relative standard deviations were ranging between 2.26% and 5.29% while the inter-day relative standard deviations were altered from 2.38% to 7.02%.

## 3. Discussion

Pyrrole is a five-membered heterocyclic ring compound containing a soliton electron at the nitrogen atom. The most widely accepted mechanism for chemical oxidative polymerization of pyrrole is the coupling between radical cations [43,48]. Specifically, the pyrrole monomer will lose an electron under the action of an oxidant and become a cationic free radical. Two cationic free radicals will combine together by collation to form a dipyrrole; similar reactions will repeat to take place and form PPy polymers [43,48]. PPy is a high-nitrogen containing polymer and is also positively charged [49]. Modification of nylon 6 membrane by PPy greatly increases the binding sites of the nanofibers. In addition, PPy can combine with different molecules in a variety of ways.

**Scheme 1:**
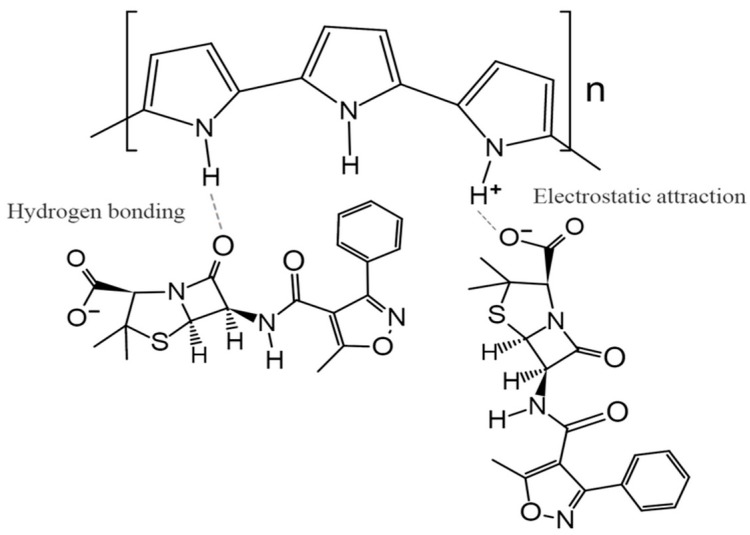
A mechanism proposed for PPy-PA6-NFsM absorbing the two β-lactams.

The mechanism by which PPy-PA6-NFsM absorbs these two antibiotics is ascribed possibly to the electrostatic attraction and to the hydrogen bonding as depicted in Scheme 1 (with oxacillin as an example). Hydrogen bonding could play a role in the adsorption. The N-H-group on the nitrogen-containing heterocyclic pyrrole is a hydrogen bond donor, but pyrrole does not contain a built-in hydrogen bond acceptor. Thus, pyrrole can be used to support hydrogen bond interactions under certain conditions [50]. The basic structure of penicillin is a thiazolidine ring attached to a β-lactam ring [51]. There are introduced substituents with greater steric hindrance at the position of side-chain amide in oxacillin. Oxacillin has hydrogen bond receptor groups on its molecular structure, such as -C=O group. Hydrogen bonding occurs via C=O···HN interactions [50]. Therefore, when the intensity of electrostatic attraction is weakened, PPy-PA6-NFsM still has a certain adsorption effect on oxacillin by the hydrogen bond.

The extent of ionization of a compound in solution is associated with pH values of the solution and the dissociation constant (pKa) of that compound [51]. When the solution pH is lower than pKa, the compound is protonated and positively charged; when the solution pH is larger than pKa, the compound is deprotonated and negatively charged. The pKa of PPy is about 10, while the pKa of oxacillin is about 2–3 [51,52]. Under solution pH between 3.0 and 6.0, the surface of the PPy is positively charged, while oxacillin is negatively charged. Due to the electrostatic interactions, higher extraction efficiency can be reached [53]. When the solution gradually turns to neutral and basic conditions, the negative charge of oxacillin gradually increases, while the positive charges on the surface of PPy-PA6-NFsM gradually decrease. These weaken the electrostatic attraction between PPy-PA6-NFsM and oxacillin. As a consequence, the adsorption amount of oxacillin on the surface of PPy-PA6-NFsM will decrease.

## 4. Materials and Methods

### 4.1. Materials

OXA and CLOX were purchased from the National Institute for the Control of Pharmaceutical and Biological Products (Beijing, China). Stock solutions (1.0 mg/mL) were prepared by dissolving a suitable amount of the antibiotics in pure water. Pyrrole and m-cresol were purchased from Aikeda Reagent Company (Chengdu, China). Nylon 6 (PA6) was purchased from Xinhui Meida Nylon Company (Guangdong, China). Borax and sodium dodecyl sulfate (SDS) were purchased from Sinopharm Chemical Reagent Company (Shanghai, China). Formic acid and ammonia were obtained from Bodi Chemical Company (Tianjin, China), which were used to adjust the pH of solutions. MeOH, EtOH, and ACN were obtained from Honeywell Burdick & Jackson (Morris, NJ, USA). All reagents were of analytical reagent grade and were employed without further purifications. Doubly distilled water was used to prepare all solution. Solutions of 0.10 M borax and 0.10 M SDS were used to prepare buffer solutions for CE-DAD analysis. Stock solutions and standards were stored in a refrigerator at 4 °C and protected from light.

### 4.2. Instrumentation

The CE instrument (P/ACE MDQ, Beckman, Brea, CA, USA) was equipped with a diode array detector, and a chromatographic workstation. An electrostatic spinning machine (Yongkang Leye, Beijing, China) was used to prepare PA6-NFsM. A scanning electron microscope (SEM, S-4800I, Hitachi, Japan) and Fourier transform infrared spectrometer (Nicolet iS, Thermo Fisher, Waltham, MA, USA) were employed to characterize PA6 and PA6-PPy-NFsM.

### 4.3. Preparation of PPy-PA6-NFsM

**Scheme 2:**
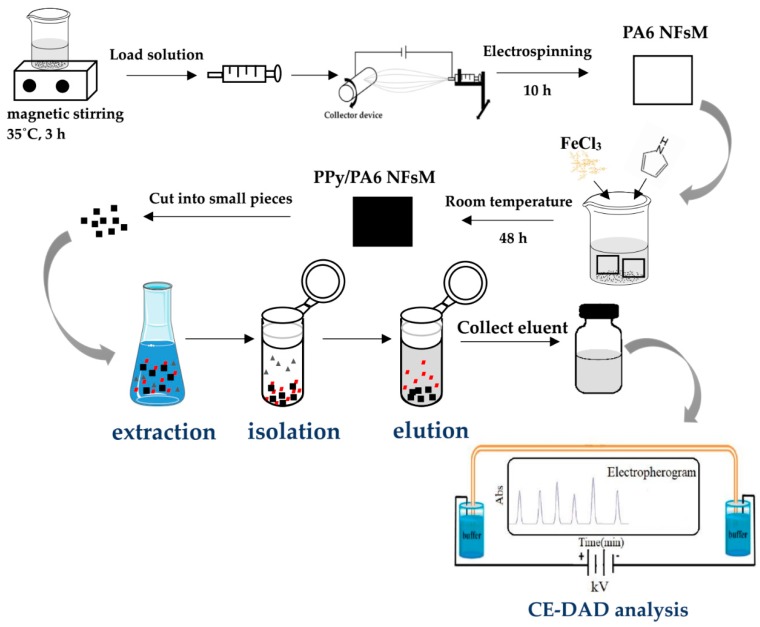
The scheme of preparation of PPy/PA6-NFsM and extraction process of the β-lactams.

The procedure, as shown in Scheme 2, was as follows. PA6 (3.0 g) was dissolved in a mixture of 6.0 mL formic acid and 4.0 mL m-cresol with magnetic stirring at 35 °C for 3 h. For the electrospinning set-up, the needle was set at a 10 cm distance from the drum collector, the ejection rate was set at 0.005 mm/min, and the applied voltage was kept at 20 kV. After 10 h of electrospinning, PA6-NFsM was obtained. Fifty milligrams of PA6-NFsM obtained above were soaked in a 20.0 mL anhydrous EtOH solution containing 0.50 mL pyrrole monomer and 0.25 g FeCl_3_·6H_2_O, then incubated for 48 h at room temperature affording crude PPy-PA6-NFsM. The crude material was washed with anhydrous ethanol and deionized water repeatedly until the solution became colorless. Finally, it was dried in an oven for 12 h at 60 °C.

### 4.4. Solid Phase Membrane Extraction Procedure

The SPE procedure as shown in Scheme 2 was: (1) PPy-PA6-NFsM (10.0 mg) was cut into small pieces (about 2–3 mm in diameter or size). (2) These small pieces were added to 20.0 mL of a water solution containing a certain amount of β-lactams (or a 40.0 mL of water sample). (3) The pH of the solution was adjusted to 4.0 using formic acid. After vigorous stirring for 30 min, the small pieces of PPy-PA6-NFsM were isolated by centrifugation at 5000 rpm for 5 min. The β-lactams were eluted from the adsorbent by 2.0 mL of a mixture of 2% ammonia in ACN. (4) After desorption, the eluent was separated from the solid parts by centrifugation at 5000 rpm for 5 min and was then evaporated to dryness under a nitrogen stream. The residues were re-dissolved in 0.25 mL water and filtered through a 0.22 μm microporous membrane (MPM) filter. (5) The sample was analyzed by the CE-DAD (vide infra).

### 4.5. CE-DAD Analysis

The electrophoretic separation was carried on the CE system. An uncoated fused quartz capillary (50 µm, 60.2 cm, the effective length was 50 cm) was used for separation at 25 °C. The running buffer consisted of 60.0 mM borax and 15.0 mM SDS (pH 9.0). The detection wavelength was set at 200 nm and the separation voltage was set at 20 kV. After 10 s injection at 0.5 psi, OXA and CLOX can be separated completely in 13 min.

### 4.6. Sample Preparations

Water samples were collected from Minxin River (Shijiazhuang, China), which were filtered through 0.22 μm MPM filters and stored in amber bottles at 4 °C in a refrigerator. The two β-lactams were directly spiked into 100.0 mL of water samples covering the range 5.0–250.0 ng/mL. A portion of 10.0 mg of the pieced PPy-PA6-NFsM was put into a 40.0 mL sample. Then, the extraction was carried out using the solid phase membrane extraction procedure described above.

## 5. Conclusions

In this work, PPy-PA6-NFsM was firstly synthesized, which was then used for a solid phase membrane for extracting OXA and CLOX in water samples before capillary electrophoresis analysis. PPy-PA6-NFsM has shown higher extraction ability toward these two antibiotics due to the electrostatic attraction and hydrogen bonding as discussed above. The LOD obtained with PPy-PA6-NFsM extraction was 2.0 ng/mL, while the LOD obtained without extraction was 0.1 μg/mL. Consequently, the solid-phase extraction makes the detection limits much lower and the measuring sensitivity much higher than a direction measurement by CE-DAD without extraction. On the other hand, these results indicate that the coating of polypyrrole on certain solid-phase carriers may form new adsorbents for extracting different antibiotics. Currently, we are planning to coat polypyrrole on other carriers to obtain a simpler but more convenient extraction process.

## Figures and Tables

**Table 1:** Method characteristics of PPy-PA6-NFsM when combined with CE-DAD. LOD: limit of detection; LOQ: limit of quantification.

Analyte	Linear Range (ng/mL)	Regression Equation	R^2^	LOD (ng/mL)	LOQ (ng/mL)
**CLOX**	5.0–250.0	Y = 251.32X − 417.45	0.9973	2.0	5.0
**OXA**	5.0–250.0	Y = 312.26X − 906.2	0.9968	2.0	5.0

**Table 2:** Comparison of various method for the determination of the two β-lactams.

Method	Sample	LOD	Founded	Recovery (%)	Reference
C18 SPE- LC–MS/MS	surface water	1.0–1.3 ng/L	n. d. ^a^	86–95	[44]
MAX SPE- HPLC-DAD	wastewater	2.8–11.2 ng/mL	- ^b^	77–96	[45]
HLB SPE-HPLC/MS/MS	river, wastewater	8 ng/L river 8–14 ng/L wastewater	OXA 10 ng/L, CLOX 15 ng/L in wastewater	83.9–95.9	[46]
HLB SPE-CE-MS/MS	river	0.26 ng/mL	-	89–96	[47]
PPy-PA6-NFsM SPE-CE-DAD	urban river	2.0 ng/mL	n. d.	84.2–96.4	This work

^a^ Not detected; ^b^ not given in text.

**Table 3:** Determination of the two β-lactams and their recoveries from river water samples.

Analyte	Added (ng/mL)	Sample 1		Sample 2		Sample 3		Intra-day RSD (%, n = 5)	Inter-day RSD (%, n = 5)
		Founded (ng/mL)	Recovery (%)	Founded (ng/mL)	Recovery (%)	Founded (ng/mL)	Recovery (%)		
**CLOX**	-	n.d. ^a^	-	n.d.	-	n.d.	-	-	-
	50.0	48.2	96.4	46.8	93.6	46.1	92.2	5.02	2.99
	100.0	87.1	87.1	89.1	89.1	84.9	84.9	5.29	4.77
	150.0	137.1	91.4	141.0	94.0	129.6	86.4	4.10	2.75
**OXA**	-	n.d.	-	n.d.	-	n.d.	-	-	-
	50.0	46.5	93.0	46.2	92.4	47.6	95.2	3.05	3.10
	100.0	84.2	84.2	86.3	86.3	89.3	89.3	2.26	2.38
	150.0	128.8	85.9	133.8	89.2	130.8	87.2	4.11	7.02

^a^ Not detected.
